# Two-dimensional perfusion angiography permits direct visualization of redistribution of flow in hepatocellular carcinoma during b-TACE

**DOI:** 10.1007/s11547-024-01816-9

**Published:** 2024-04-18

**Authors:** Pierleone Lucatelli, Simone Ciaglia, Bianca Rocco, Gianluca De Rubeis, Guido Bolognesi, Elio Damato, Mario Corona, Pier Giorgio Nardis, Alessandro Cannavale, Paolo Ricci, Carlo Catalano

**Affiliations:** 1 Unit of Vascular and Interventional Radiology, Department of Radiological Oncological and Anatomo-Pathological Sciences, Sapienza University of Rome, Viale del Policlinico 155, 00161 Rome, Italy; 2 Department of Diagnostic, UOC of Diagnostic and Interventional Neuroradiology, San Camillo-Forlanini Hospital, Rome, Italy; 3 Department of Chemical Engineering, Loughborough University, Loughborough, LE11 3TU UK; 4 Department of Chemistry, University College London, London, WC1H 0AJ UK; 5 Unit of Emergency Radiology, Policlinico Umberto I Hospital, Sapienza University of Rome, Viale del Policlinico 155, 00161 Rome, Italy; 6 Department of Radiological Sciences, Oncology and Pathology, Policlinico Umberto I Hospital, Sapienza University of Rome, Viale del Policlinico 155, 00161 SapienzaRome, Italy

**Keywords:** DEM-TACE, b-TACE, Hepatocellular carcinoma, Two-dimensional perfusion angiographies, Balloon microcatheter, In silico model

## Abstract

**Objectives:**

To demonstrate in vivo redistribution of the blood flow towards HCC’s lesions by utilizing two-dimensional perfusion angiography in b-TACE procedures.

**Material and methods:**

In total, 30 patients with 35 HCC nodules treated in the period between January 2019 and November 2021. For each patient, a post-processing software leading to a two-dimensional perfusion angiography was applied on each angiography performed via balloon microcatheter, before and after inflation. On the colour map obtained, reflecting the evolution of contrast intensity change over time, five regions of interests (ROIs) were assessed: one on the tumour (ROI-t), two in the immediate peritumoural healthy liver parenchyma (ROI-ihl) and two in the peripheral healthy liver parenchyma (ROI-phl). The results have been interpreted with a novel in silico model that simulates the hemodynamics of the hepatic arterial system.

**Results:**

Among the ROIs drawn inside the same segment of target lesion, the time-to-peak of the ROI-t and of the ROI-ihl have a significantly higher mean value when the balloon was inflated compared with the ROIs obtained with deflated balloon (10.33 ± 3.66 s vs 8.87 ± 2.60 s (*p* = 0.015) for ROI-t; 10.50 ± 3.65 s vs 9.23 ± 2.70 s (*p* = 0.047) for ROI-ihl). The in silico model prediction time-to-peak delays when balloon was inflated, match with those observed in vivo. The numerical flow analysis shows how time-to-peak delays are caused by the obstruction of the balloon-occluded artery and the opening of intra-hepatic collateral.

**Conclusion:**

The measurements identify predictively the flow redistribution in the hepatic arteries during b-TACE, supporting a proper positioning of the balloon microcatheter.

**Supplementary Information:**

The online version contains supplementary material available at 10.1007/s11547-024-01816-9.

## Introduction

Trans-arterial chemoembolization (TACE) is the treatment of choice for the intermediate stage (Stage B) and early and very early-stage hepatocellular carcinoma (HCC) when curative treatments are not feasible [1–3]. Drug-eluting microsphere TACE (DEM-TACE) with the use of balloon microcatheter for temporary artery occlusion (b-TACE), has been proposed as a useful tool to improve drug deposition [4], leading to an improved treatment response [5–7] over non-occluded procedures. The underlying mechanism permitting flow restoration beyond the temporary occluded segment, is the opening of intra-hepatic collaterals [8]. This compensation mechanism has been also observed in in silico zero-dimensional and three-dimensional hepatic artery hemodynamics models by Aramburu et al. [9–11]. However, such numerical models are limited to the blood flow analysis only, and no numerical investigation of the distribution and accumulation of blood additives (e.g. embolic materials, contrast agents) has been reported to date. The current interpretation of the compensation mechanism is corroborated by the existing hemodynamics models [9–11], and it is in agreement with in vivo pressure measurements after temporary occlusion. However, to date post-procedural assessment of the b-TACE benefit has been reported in vivo only [12] and no radiological tools are known that allow a direct predictive assessment of flow redistribution.

Two-dimensional perfusion angiography (2DPA) provides a quantitative evaluation of tissue perfusion by creating a 2D colour map of intrahepatic arteries corresponding to mean transit time of contrast media agent, and a time density curve (TDC), even in retrospective fashion from the digital subtraction angiography (DSA). Therefore, 2DPA may be useful in determining the occurrence of redistribution of flow during b-TACE procedures. This technique has a limited literature evidence, being mainly investigated for the identification of predicting factors for critical limb ischemia revascularization success [13–15]. As for liver embolization procedures, 2DPA has been adopted to demonstrate how the reduction of tumour perfusion can predict TACE success [16–18].

The aim of our study is to retrospectively evaluate if 2DPA is a useful tool to assess in vivo the redistribution of the flow towards the HCC’s nodules. This was performed by acquiring 2DPA measurements—before and immediately after temporary occlusion during b-TACE procedures—to determine how the inflation of the microballoon affects the time required by the contrast media agent to travel from the microcatheter tip to the predefined regions of interest (ROIs) of the hepatic artery system—a time hereby referred as peak time of the ROIs. To interpret the in vivo observations of the ROI peak time alterations induced by the inflated microballoon, we developed a new in silico model for b-TACE. This model can describe both the blood flow distribution as well as the transport and accumulation of blood additives in hepatic arteries, which is necessary for predicting the ROI peak times. Finally, by comparing the measured and simulated ROI peak times, we could assess the extent of the in vivo redistribution of the flow towards the HCC’s nodules.

## Materials and methods

This retrospective and observational single-centre study was approved by the ethical review board of our institution (RIF 6072). Informed consent for the procedure and for anonymized publication of non-sensitive data was obtained from all individual patients included in the study. Inclusion criteria were only those patients in which super-selective angiography via the balloon microcatheter was performed immediately before and after inflation. TACE indications were discussed in the multidisciplinary tumour board (comprising a transplant surgeon, an interventional radiologist, body radiologist and a hepatologist).

### B-TACE procedure

All b-TACE procedures were performed according to CIRSE standard of practice [19] by experienced interventional radiologists (> 10 years). After having positioned a 4 Fr. catheter in the hepatic proper artery, detailed liver vasculature map was obtained via digital subtraction angiography and dual-phase cone beam CT (CBCT). After individuation of the feeding artery, superselective catheterization was performed with a 2.8 Fr balloon microcatheter Occlusafe^®^ (Terumo, Tokyo, Japan) [20]. Once the balloon micro-catheter was positioned, DSA was performed before and after balloon inflation.

The embolization protocol was highly standardized, consisting in a sequential embolization using 100 ± 25 µm PEG (Lifepearl^®^—Terumo, Tokyo, Japan) microspheres eventually followed, if stasis was not reached, by 200 ± 50 µm PEG microspheres. Both microspheres are each pre-loaded with 50 mg of doxorubicin [21]. The endpoint of b-TACE was either the upstream reflux of microspheres despite balloon inflation, or the inversion of flow in the vascular anastomosis that restored flow beyond the temporary occluded segment (potential non-target embolization), or manual perception of increased resistance during injection, or the administration of the maximum threshold of drug.

### Image analysis

**Fig. 1:**
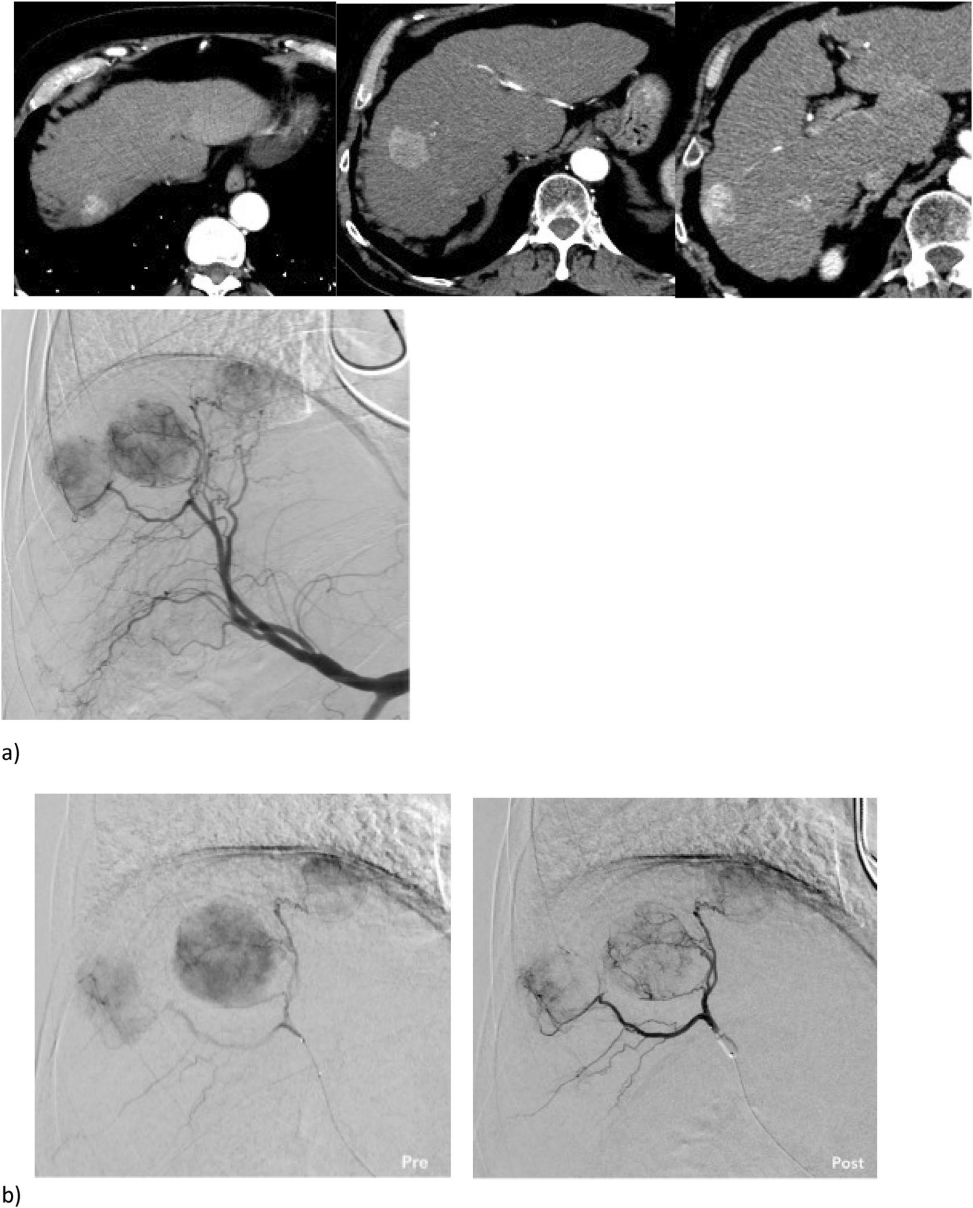

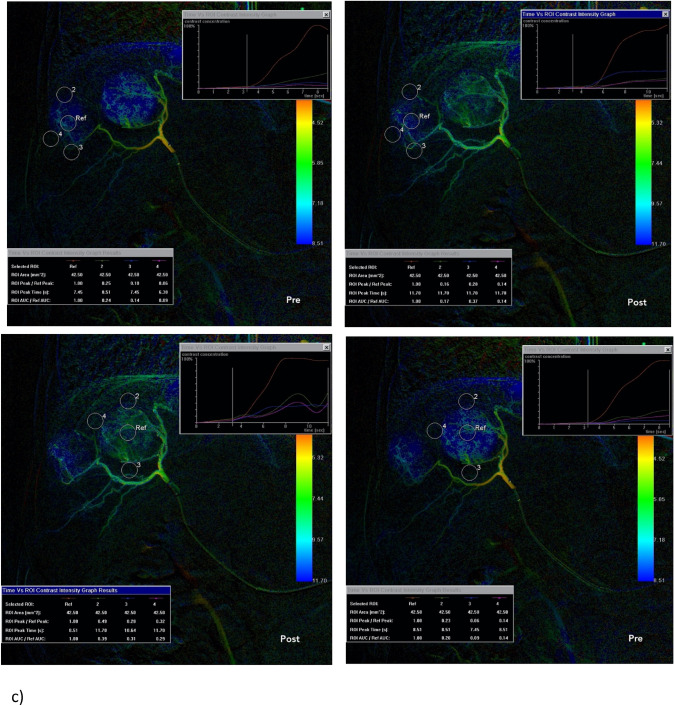
**a** CT scans showing three hypervascular HCC nodules located in segment 8 and 7 as confirmed by selective angiography performed in the common hepatic artery. **b** Superselective catheterization performed with balloon microcatheter Occlusafe^®^ (Terumo, Japan). The angiography performed with inflated balloon (post) detects more feeding vessels compared to angiography performed with deflated balloon (pre). **c** Syngo iFlow is applied to selective angiographies, performed before embolization with both deflated and inflated micro-balloon, showing an increase of peak time in the ROI-t located in the centre of tumour and the ROI-ihl located in the healthy parenchyma of the same segment

DSA data were transferred on a dedicated workstation (syngo X-Workplace VB21: Siemens Healthineers, Erlangen—Germany), and for each treated patient Syngo iFlow (Siemens Healthineers, Erlangen—Germany) post-processing software was applied on angiography performed via coaxial balloon microcatheter, immediately before and after micro-balloon inflation. The software elaboration leads to a bidimensional colour map, reflecting the evolution of contrast intensity change over time. Then a coded specific colour map is generated, ranging from red (early maximum intensity) to blue (late maximum intensity). On this map is possible to draw regions of interest. On each acquired angiography, the following five different regions of interests (ROIs) were assessed: one ROI in the tumour (ROI-*t*), two distinct ROIs in the immediate peritumoural healthy liver parenchyma (ROI-*ihl*), other two distinct ROIs in the peripheral healthy liver parenchyma in the enhanced segment (ROI-*phl*). As shown in Fig. 1, for each ROI the software calculated the area (mm^2^), peak time ratio and AUC (area under the time density curve).

### In silico model

**Fig. 2:**
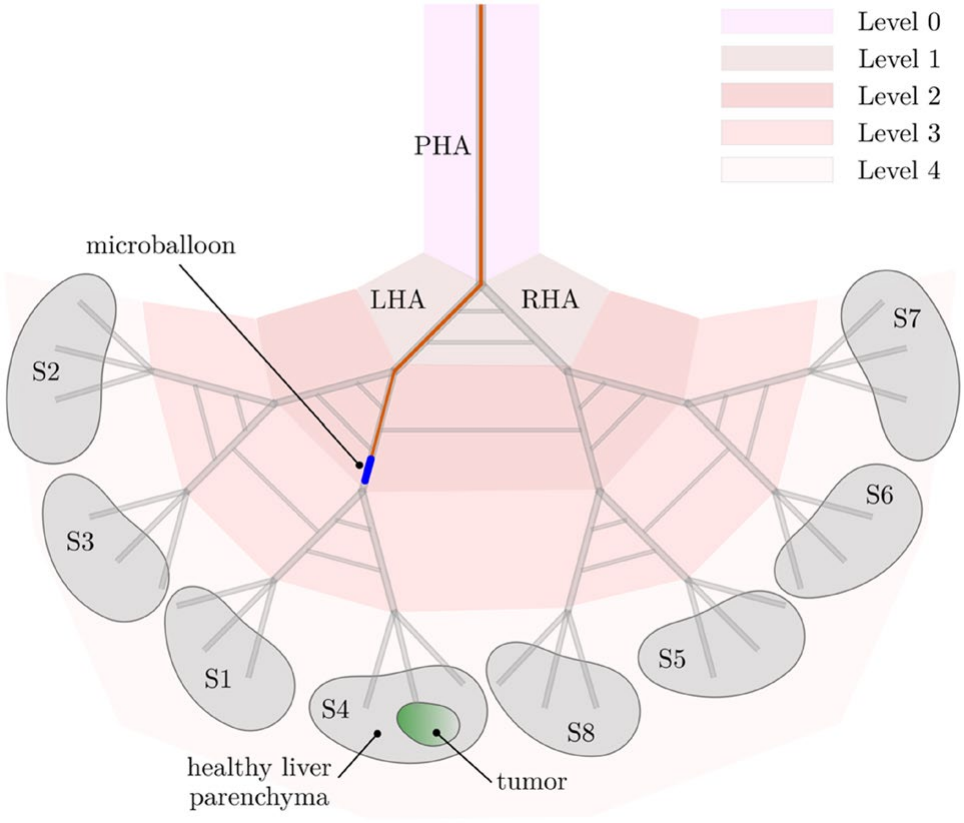
Network of interconnected artery branches used in the numerical model to describe the hepatic arterial system. The artery system is split into levels whose boundaries are located at the artery branch bifurcations. The proper hepatic artery (Level 0) bifurcates into the left hepatic artery and right hepatic artery (Level 1), which further splits into four daughter arteries (Level 2), eight segmental arteries (Level 3) and twenty-four sub-segmental arteries (Level 4). At each artery bifurcation, two communicating arcades join the diverging artery branches downstream of the bifurcation. Communicating arcades joining the daughter arteries (Level 2), the segmental arteries S3 and S1 (Level 3) and the segmental arteries S5 and S6 (Level 3) are also included. The tumour is located in segment S4 and the balloon is positioned in the left daughter artery feeding the segments S1 and S4

The hemodynamics of the hepatic arterial system—from the proper hepatic artery to the segmental arteries—was simulated numerically via the zero-dimensional (0D) model introduced by Aramburu et al. [9–11]. A detailed description of the model is provided in the Supporting Information (SI) document. The hepatic artery is described by a network of interconnected artery branches, which resembles closely the one proposed by Aramburu et al. [9–11]. As shown in Fig. 2, the branch network connects the proper hepatic artery to the liver segments, S1-8, and the tumour is located in segment S4. To mimic the in vivo b-TACE conditions, the balloon microcatheter is placed at the end of the daughter vessel that is feeding the segmental arteries S1 and S4. When the micro-balloon is inflated, the blood flow in the artery is obstructed. The transport of dye in the artery system was modelled by solving the time-dependent convection–diffusion equation for the dye concentration and applying a mass balance at the nodes of the artery branch network. A full description of the model is provided in the SI document.

### Statistical analysis

Normal distribution was tested using Kolmogorov–Smirnov Z; continuous variable was reported accordingly. Paired T test groups were used for comparing parameters derived from iFlow software (Siemens, Enrlargen) in the ROIs between inflated and deflated balloon microcatheter. The Bland and Altman plot was added for visualization. Furthermore, BladP < 0.05 was deemed as statistically significant. MedCalc 18.2.1 (MedCalc, Mariakerke, Belgium) was used as statistical software.

## Results

### 2DPA analysis

**Fig. 3:**
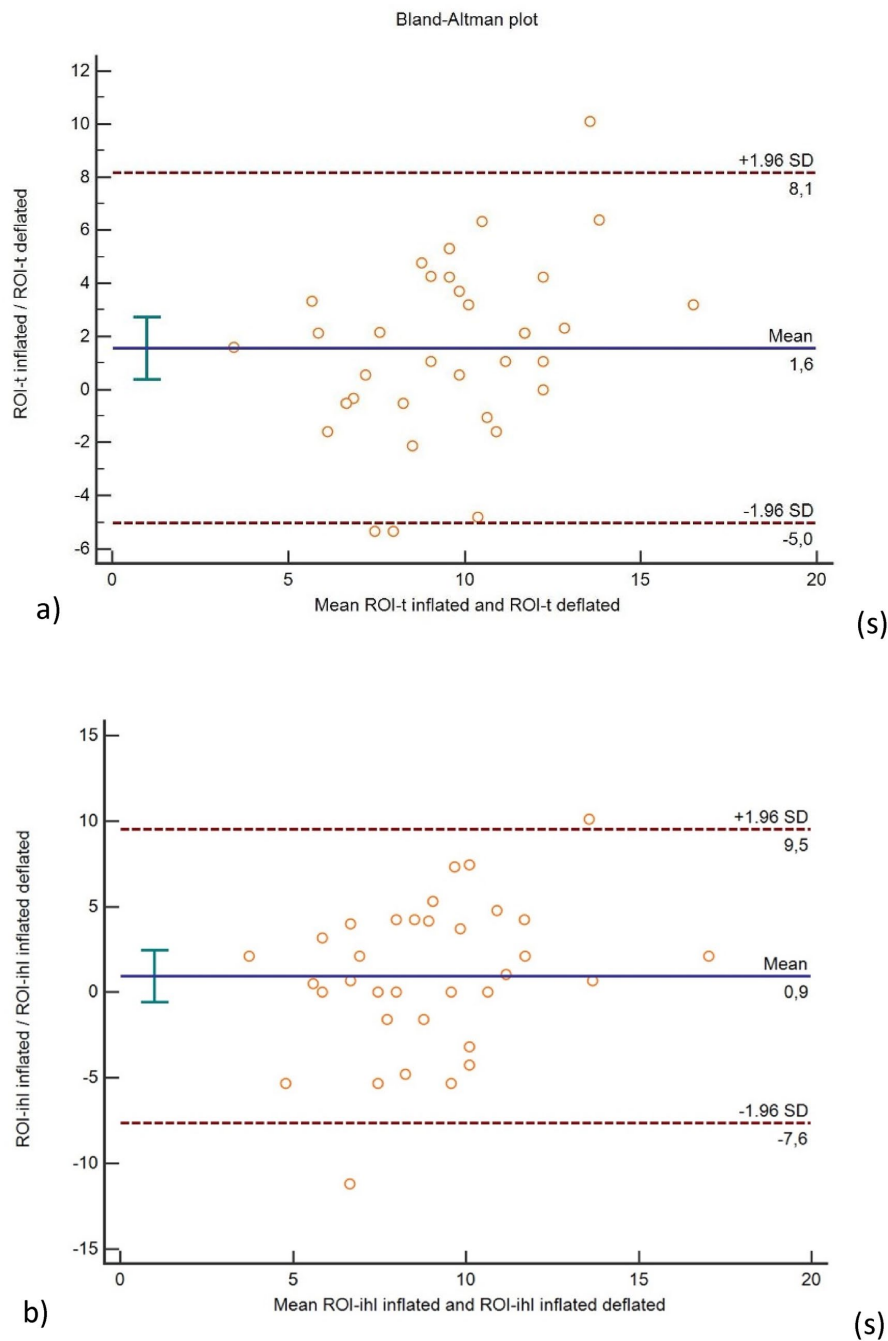
Bland–Altman plot ROI-t (**a**) and ROI-ihl (**b**). ROI-t: region of interest-tumour; ROI-ihl: region of interest-immediate peritumoural liver parenchyma; SD: standard deviation. The plot compared the differences of each measurement versus the mean of the measurements and standard deviation

The data of 33 patients with 40 HCC tumours treated in our centre in a period between January 2019 and November 2021 were reviewed, and the final population enrolled in this study encountered 30 patients with 35 treated HCCs. Among the ROIs drawn inside the same segment of the target lesion treated with b-TACE, the peak time of the tumour ROI (ROI-t) and the parenchyma ROI (ROI-ihl) have a significantly higher mean value when the balloon microcatheter was inflated comparing with the ROIs obtained with deflated micro-balloon (10.33 ± 3.66 s vs 8.87 ± 2.60 s (*p* = 0.015) for ROI-t; 10.50 ± 3.65 s vs 9.23 ± 2.70 s (*p* = 0.047) for ROI-ihl), as shown in Fig. 3. No other values were statistically significant (see Table 1). The higher mean value of the peak time of ROI-t and ROI-ihl, reflects the longer time of contrast agent to reach the HCC’s nodules and the immediate peritumoural healthy parenchyma when the balloon is inflated. This result is expected as the inflated balloon obstructs the blood flow, thus causing a delay in the transport of the contrast agent. The extent of the delay is quantified by the mean peak time differences between deflated and inflated balloon microcatheter, which were 1.46 ± 3.36 s for ROI-t and 1.26 ± 3.62 s (*p* = 0.81) for ROI-ihl. These correspond to an increase of the peak time—namely a relative peak time delay—of 16.6% compared to 13.7%, respectively. Regarding the ROIs taken in the peripheral healthy parenchyma in the same enhanced segments of the target lesion (ROI-phl), no statistically significant differences among all parameters with inflated and deflated balloon microcatheter were observed (see Table 2). This suggests that the blood flow obstruction, induced by the inflated balloon, has a negligible effect on the transport of the contrast agent to the peripheral healthy parenchyma.

**Table 1:** Immediate peritumoural healthy liver parenchyma (ihl)

		n	Inflated		Deflated		Paired differences			
			Mean (s)	SD	Mean (s)	SD	Mean (s)	SD	95% CI	P^a^
IHL- parenchyma	PEAK TIME ROI—T	35	10.33	3.66	8.87	2.60	1.46	3.36	0.31 to 2.61	**0.015**
	PEAK TIME ROI 2	35	10.50	3.65	9.23	2.70	− 1.26	3.62	− 2.51 to − 0.02	**0.047**
	PEAK TIME ROI 3	35	10.43	3.51	9.22	2.69	− 1.20	3.49	− 2.39 to 0.0021	0.05
	PEAK TIME ROI 4	33	34.18	137.17	63.45	313.37	29.27	344.56	− 92.90 to 151.45	0.63
Peak time ratio	$\frac{{{\text{ROI }}2{\text{ PEAK TIME IHL }}}}{{\text{ROI PEAK TIME T }}}$	35	0.55	0.23	0.56	0.36	− 0.01	0.40	− 0.14 to 0.13	0.93
	$\frac{{{\text{ROI }}3{\text{ PEAK TIME IHL }}}}{{{\text{ROI }}3{\text{ PEAK TIME T}}}}$	35	0.56	0.25	0.56	0.25	0.004	0.30	− 0.09 to 0.11	0.93
	$\frac{{{\text{ROI }}4{\text{ PEAK TIME IHL }}}}{{\text{ROI PEAK TIME T}}}$	33	0.60	0.40	0.51	0.22	− 0.09	0.43	− 0.24 to 0.06	0.23
AUC ratio	$\frac{{{\text{ROI }}2{\text{ AUC IHL }}}}{{\text{ROI AUC T }}}$	35	0.55	0.22	0.54	0.36	0.01	0.37	− 0.12 to 0.14	0.84
	$\frac{{{\text{ROI }}3{\text{ AUC IHL }}}}{{\text{ROI AUC T }}}$	35	0.59	0.26	0.54	0.30	− 0.05	0.34	− 0.17 to 0.06	0.36
	$\frac{{{\text{ROI }}4{\text{ AUC IHL }}}}{{\text{ROI AUC T }}}$	33	0.52	0.29	0.47	0.20	− 0.06	0.30	− 0.16 to 0.05	0.29

Bold values indicate the significant p values (< 0.05)

^a^Paired samples *t* test

**Table 2:** Peripheral healthy liver parenchyma (phl)

		n	Inflated		Deflated		Paired differences			
			Mean (s)	SD	Mean (s)	SD	Mean (s)	SD	95% CI	P ^a^
PHL- parenchyma	PEAK TIME ROI T	35	10.13	3.61	8.93	2.68	− 1.20	3.82	− 2.51 to 0.11	0.07
	PEAK TIME ROI 2	35	9.37	3.93	31.03	133.49	21.66	133.76	− 24.29 to 67.61	0.34
	PEAK TIME ROI 3	35	9.19	4.28	8.69	3.01	− 0.50	4.46	− 2.03 to 1.04	0.52
Peak time ratio	$\frac{{{\text{ROI }}2{\text{ PEAK TIME PHL }}}}{{\text{ROI PEAK TIME T}}}$	35	0.37	0.26	0.32	0.24	− 0.04	0.36	− 0.17 to 0.08	0.46
	$\frac{{{\text{ROI }}3{\text{ PEAK TIME PHL }}}}{{\text{ROI PEAK TIME T }}}$	35	0.34	0.23	0.29	0.21	− 0.05	0.26	− 0.13 to 0.04	0.30
AUC ratio	$\frac{{{\text{ROI }}2{\text{ AUC PHL }}}}{{\text{ROI AUC T }}}$	35	0.39	0.33	0.32	0.23	− 0.07	0.38	− 0.20 to 0.06	0.26
	$\frac{{{\text{ROI }}3{\text{ AUC PHL }}}}{{\text{ROI AUC T }}}$	35	0.37	0.29	0.31	0.22	− 0.06	0.32	− 0.17 to 0.05	0.25

To summarize, 2DPA measurements enabled the identification of the regions where the inflated balloon affected the blood flow and, thus, the transport of the contrast agent. However, such measurements alone do not allow us to confirm in vivo the role of flow redistribution and opening of intra-hepatic collaterals. To do so, an in silico model for b-TACE, which can simulate both the arterial hemodynamics and the transport of blood additives (e.g. contrast agent), is needed to correlate the peak time delays with the opening of intra-hepatic collaterals and the flow redistribution in the tumour and adjacent regions.

### In silico analysis

**Fig. 4:**
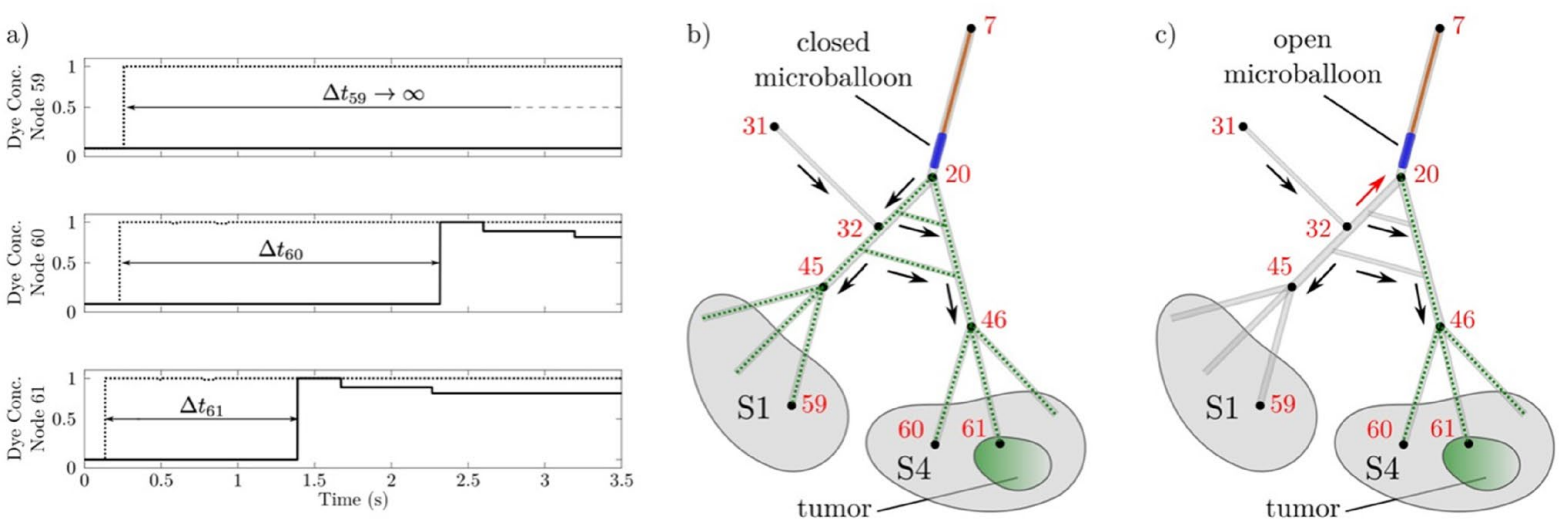
**a** Time evolution of the dye concentration, $c$, at node 59 (healthy parenchyma in segment S1), 60 (healthy parenchyma in segment S4), 61 (tumour) with deflated (dashed lines) and inflated (solid lines) balloon. **b**, **c** Schematics of the hepatic artery system downstream the balloon catheter. Arrows shows the blood flow direction in arteries. Dashed lines show the trajectories of dye ejected from the catheter. When the balloon catheter is open (**c**), the blood flood in artery branch 20–32 is reversed (red arrow) and dye reaches S4 only

Figure 4a shows the time evolution of the dye concentration at three nodes: the adjacent healthy parenchyma in segment S1 (node 59), the immediate peritumoural healthy parenchyma (node 60) and the tumour (node 61) in segment S4. When the balloon is deflated, the peak time at the tumour ($t_{61,{\text{peak}}} = 0.14$ s) is shorter than the peak time at the immediate peritumoural ($t_{60,{\text{peak}}} = 0.23$ s) and adjacent ($t_{59,{\text{peak}}} = 0.26$ s) healthy parenchyma, as expected due to lower downstream vasculature resistance at the tumour location compared to the healthy parenchyma. The pressure at the catheter tip is 89.18 mmHg, which is close to the proximal artery pressure 93.30 mmHg.

Crucially, the inflation of the balloon causes an increase of peak times—namely, a peak time delay—at both the tumour ($$t_{61,{\text{peak}}} = 1.39\; {\text{s}}$$) and peritumoural healthy parenchyma ($$t_{60,peak} = 2.32$$ s). The peak time differences between inflated and deflated balloon are $$\Delta t_{61} = 1.25 $$ s for the tumour and $$\Delta t_{60} = 2.09 $$ seconds for the peritumoural healthy parenchyma. These values compare very well with the in vivo results (i.e. 1.46 ± 3.36 s for the tumour and 1.26 ± 3.62 s for the peritumoural healthy parenchyma). Note that in the model the peak time delay for the peritumoural healthy parenchyma is larger than the one for the tumour. This is due to the lower hydrodynamic resistance of the tumour-feeding artery (branch 46–61) compared to the parenchyma feeding arteries (branch 46–60), which results in a faster blood flow towards the tumour ($$q_{46,61} = 9.49 $$ mL/min vs $$q_{46,60} = 3.82 $$ mL/min). Such a difference is not noticeable in the in vivo average peak time data, probably due to the relatively large experimental uncertainties. The balloon‐occluded arterial stump pressure (BOASP) is 68.94 mmHg, which is close to the distal artery pressure 66.66 mmHg.

Table 3 shows the alteration of the blood flow rates, induced by the balloon inflation, at the occluded artery (node 20), the communicating arcade feeding the tumour segment S4 (node 32), the tumour-feeding artery (node 61) and the immediate peritumoural healthy parenchyma (node 60). The direction of the corresponding blood flows is shown in the schematics in Fig. 4b, c. When the balloon is deflated, the parenchyma in segments S1 and S4 are almost entirely fed by the blood stream passing through node 20, where the microcatheter is located. Indeed, the blood flow rate from the communicating arcade (node 32) is negligible—namely, less than 0.1% of the flow rate through node 20. On the other hand, when the balloon is inflated, the blood stream to segments S1 and S4 is supplied entirely by the communicating arcade, since the artery at node 20 is fully occluded by the balloon. Notably, the increased hydrodynamic resistance induced by the occluding balloon generates a substantial drop of the rate of blood flow reaching segments S1 and S4, from 283.5 mL min^−1^ with deflated balloon to 29 mL min^−1^ with inflated balloon. This redistribution of the flow thus explains the peak time delays observed both in vivo and in silico.

**Table 3:** Flow rates in mL min^−1^, predicted by the in silico model, at the occluded artery (node 20), intra-hepatic collateral (node 32), tumour-feeding artery (node 61) and immediate peritumoural healthy parenchyma (IPHP, node 60)

Location	Occluded artery	Collateral	Tumour	IPHP
Symbol	$q_{20}$	$q_{32}$	$q_{61}$	$q_{60}$
Deflated (closed) balloon	283.3	0.226	93.61	37.71
Inflated (open) balloon	0	29.08	9.49	3.82

**Fig. 5:**
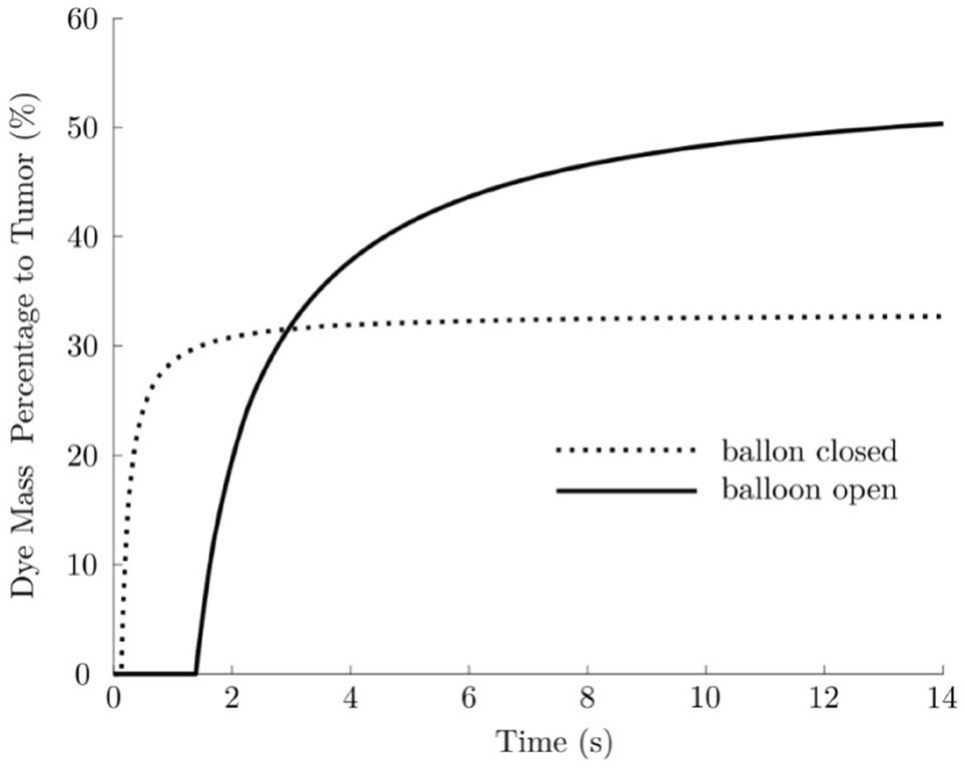
Time evolution of the percentage of dye mass, ejected from the catheter, that is delivered to tumour, when balloon is closed (dashed line) and when balloon is open (solid line). The mass percentages were calculated according to Eq. (S15) and (S16)

Furthermore, the balloon-induced occlusion of the selected vessel causes a flow reversal in the S1-feeding artery between nodes 20 and 32 (red arrow in Fig. 4c). Consequently, the dye reaches S4 only and the peak time at node 59 becomes infinite (Fig. 4a)—i.e. no dye is delivered to S1. A similar outcome was observed also in other in silico and in vitro models [9]. Interestingly, our in vivo results show that the peak time of the ROI corresponding to the peripheral healthy liver parenchyma (ROI-*phl*) always remains finite (Table 2); therefore, the contrast agent does reach the healthy parenchyma segments adjacent to the tumour segment even when the catheter is inflated. This is due to the inevitable limitations of the existing zero-dimensional in silico models in describing the actual level of collaterality of hepatic artery system. Finally, the efficacy of the b-TACE treatment is confirmed by the significant increase in the dye mass percentage delivered to the tumour (Fig. 5) caused by balloon inflation. Namely, when the balloon is closed, only 32.7% of the dye ejected from the catheter has reached the tumour after 14 s. This percentage increases to 50.4% when the balloon is open—namely a ca. 54% increase.

## Discussion

Our findings show how 2DPA detects and quantifies the blood flow redistribution after balloon occlusion in the tumour and surrounding liver parenchyma by the change of peak time before and after balloon microcatheter inflation. This real-time evaluation allows to intra-procedurally detect the correct position of balloon microcatheter. In fact, the oncological efficacy of b-TACE vs c-TACE is well established with a better complete response (71.7 vs. 54.1%, respectively [*p* = 0.047]) for tumours > 3 cm and a lower re-treat rate for all lesions (12.1 vs. 26.9%, respectively; *p* = 0.005). However, b-TACE is effective only in predeterminate hemodynamic situations [7], namely when the redistribution of blood flow occurs and it leads to the decrease of BOASP about 64 mmHg [4]. However, this parameter does not reflect completely the complexity of liver/tumour blood flow modification after the inflation of the balloon microcatheter. The iFlow software permits to evaluate the modification of the perfusion in the target area of treatment [22] before the execution of the embolization itself. This facilitates a proper positioning of the balloon microcatheter, thus finally enhancing the oncological effect of the TACE procedure. A novel zero-dimensional in silico hemodynamic model, which simulates the transport of blood additives in the hepatic artery system, was developed to confirm the correlation between peak time delays and flow redistribution to the target (tumour) region induced by the inflation of the catheter balloon. A good quantitative agreement was observed between the tumour’s and surrounding liver parenchyma’s peak time delays predicted by the numerical model and those measured in vivo via 2D perfusion angiography. The numerical study shows also that the peak time delays are caused by the obstruction of the balloon-occluded artery and the opening of the intra-hepatic collaterals. Finally, the model demonstrates how the balloon-induced flow redistribution leads to a significant increase (ca. 55%) in the amount of additive material (e.g. contrast agent, drug) delivered to target (tumour) region from the catheter—a finding that is consistent with the proven improved efficacy of the b-TACE treatment compared to conventional TACE [6, 12].

To date, the evidence of the flow redistribution caused by selective occlusion of arteries in b-TACE treatment has been limited to in vivo pressure (BOASP) measurements [19], in silico simulations of blood flow and pressure fields [10, 11], in vitro modelling of dye transport [9] and finally post-procedural evaluation with depositional analysis of embolic agent in course of B-TACE and B-SIRT [12]. In this study, we report in vivo evidence of the flow redistribution induced by the catheter balloon via 2D perfusion angiography measurements. Therefore, for the first time, the effects of flow redistribution on the transport of a contrast agent are directly observed and not only inferred through pressure drop measurements. The proposed 2D perfusion angiography procedure can allow clinicians to identify, before the execution of the embolization procedure, the appropriate device position and level of balloon inflation, which are key to the successful outcome of the b-TACE treatment. The measured peak time delays in the tumour and surrounding liver parenchyma regions are reproducible features, independent from the operator skills, and they could be therefore added to the already known tool of BOASP measurements [4], usually used to evaluate appropriate micro-balloon inflation and the occurrence of flow redistribution.

This study has some limitations. First, the nature of the study is retrospective and observational, without randomization. Second the enrolled sample size (30 patients, 35 HCC nodules) is relatively small and encompasses only hypervascular HCC nodules, thus not reflecting completely the clinical practice. As for the numerical study, a number of simplifying assumptions were adopted to reduce the complexity of the mathematical model as well as the computational cost of the simulations. These include a steady-state zero-dimensional approximation of the blood flow and additive transport, and a simplified description of the artery system geometry and collaterality as well as of the properties of blood (i.e. Newtonian fluid) and arteries (i.e. circular, rigid and straight vessels). Future work should focus on the development of advanced numerical models adopting refined descriptions of arteries, blood and flow properties (e.g. three-dimensional geometries, pulsatile flows, elastic vessels and non-Newtonian fluids) to achieve more realistic predictions of flow redistribution and drug transport in b-TACE treatment.

In conclusion, the measurement of BOASP is an indirect parameter to evaluate flow redistribution towards HCC nodules. Conversely, 2DPA stands as tool capable of directly and predictively assessing flow redistribution during B-TACE, as demonstrated by our measurements and in silico model predictions. Thus, 2DPA offers a valid aid to interventional radiologist in accurately positioning the balloon microcatheter, thereby increasing the likelihood of a successful outcome for the clinical intervention.

### Supplementary Information

Below is the link to the electronic supplementary material.

Supplementary file1 (DOCX 1714 kb)

Supplementary file2 (MOV 7849 kb)

## Abbreviations

DEM-TACE: Drug-eluting microspheres—transarterial chemoembolization
HCC: Hepatocellular carcinoma
TACE: Transarterial chemoembolization
b-TACE: Balloon-occluded—transarterial chemoembolization
BOASP: Balloon-occluded arterial stump pressure
2DPA: Two-dimensional perfusion angiography
TDC: Time density curve
DSA: Digital subtraction angiography
ROI-t: Region of interest in the tumour
ROI-phl: Region of interest on the peripheral healthy liver parenchyma
ROI-Ihl: Region of interest in the immediate peritumoural liver parenchyma
