# Supplementary material for: Two-dimensional perfusion angiography permits direct visualization of redistribution of flow in hepatocellular carcinoma during b-TACE
Source: Radiol Med. 2024 Apr 18;129(6):823–33. doi: 10.1007/s11547-024-01816-9 (PMC11169056; doi:10.1007/s11547-024-01816-9)
Supplement: Supplementary file 1 — Supplementary file1 (DOCX 1714 kb) [file 11547_2024_1816_MOESM1_ESM.docx]

**Supporting Information**

NUMERICAL METHODS

*Hemodynamics*

The hemodynamics of the hepatic arterial system – from the proper hepatic artery to the segmental arteries – was simulated numerically via a zero-dimensional (0D) model. This model adopts the hydraulic-electric circuit analogy (1) and assumes that (i) viscous effects are dominant and inertia is negligible (i.e. small Reynolds and Womersley numbers), (ii) blood is a Newtonian fluid within the examined range of shear rates, (iii) arteries are circular, rigid and straight, (iv) the flow is steady, isothermal and incompressible. Under these conditions, only the resistive effect of the arteries is considered, and the pressure drop, $\Delta P,$ within an artery branch is given by

$$\Delta P=R q$$

(S1)

with $q$ the blood flow rate in the artery and $R$ the artery hydraulic resistance. According to the Hagen-Poiseuille’s law, the resistance can be expressed as

$$R_{h}=\frac{128 \mu}{\pi}\frac{L}{d}$$

(S2)

with $\mu=0.00309 Pa s$ (2) the blood viscosity, and $L$ and $d$ the artery length and diameter, respectively.

Here, the hepatic artery is described by a network of interconnected artery branches as showed in **Figure 2**. Our representation of the hepatic artery resembles closely the one proposed by Aramburu et al. (3). The proper hepatic artery (Level 0) bifurcates into the left hepatic artery and right hepatic artery (Level 1), which further splits into four daughter arteries (Level 2), eight segmental arteries (Level 3) and twenty-four sub-segmental arteries (Level 4). The liver segments, S1-8, are defined as in Ref. 3. At each artery bifurcation, two communicating arcades – here denoted as upper and lower communicating arcade – join the diverging artery branches downstream of the bifurcation. For sake of simplicity, it is assumed that the communicating arcades intersect the branches at one-third and two-thirds along the branch length. Furthermore, we also included communicating arcades joining left and right daughter arteries (Level 2), the segmental arteries S3 and S1 (Level 3) and the segmental arteries S5 and S6 (Level 3). These communicating arcades meet the arteries at their mid-point. The geometrical properties of the hepatic artery network used in the numerical simulations are reported in **Table S1**. It is assumed that the tumour is located in segment S4 and it is fed by the central subsegmental artery of that segment. To mimic the in vivo b-TACE conditions, the balloon microcatheter is placed at the end of the daughter vessel (Level 2) that is feeding the segmental arteries S1 and S4.


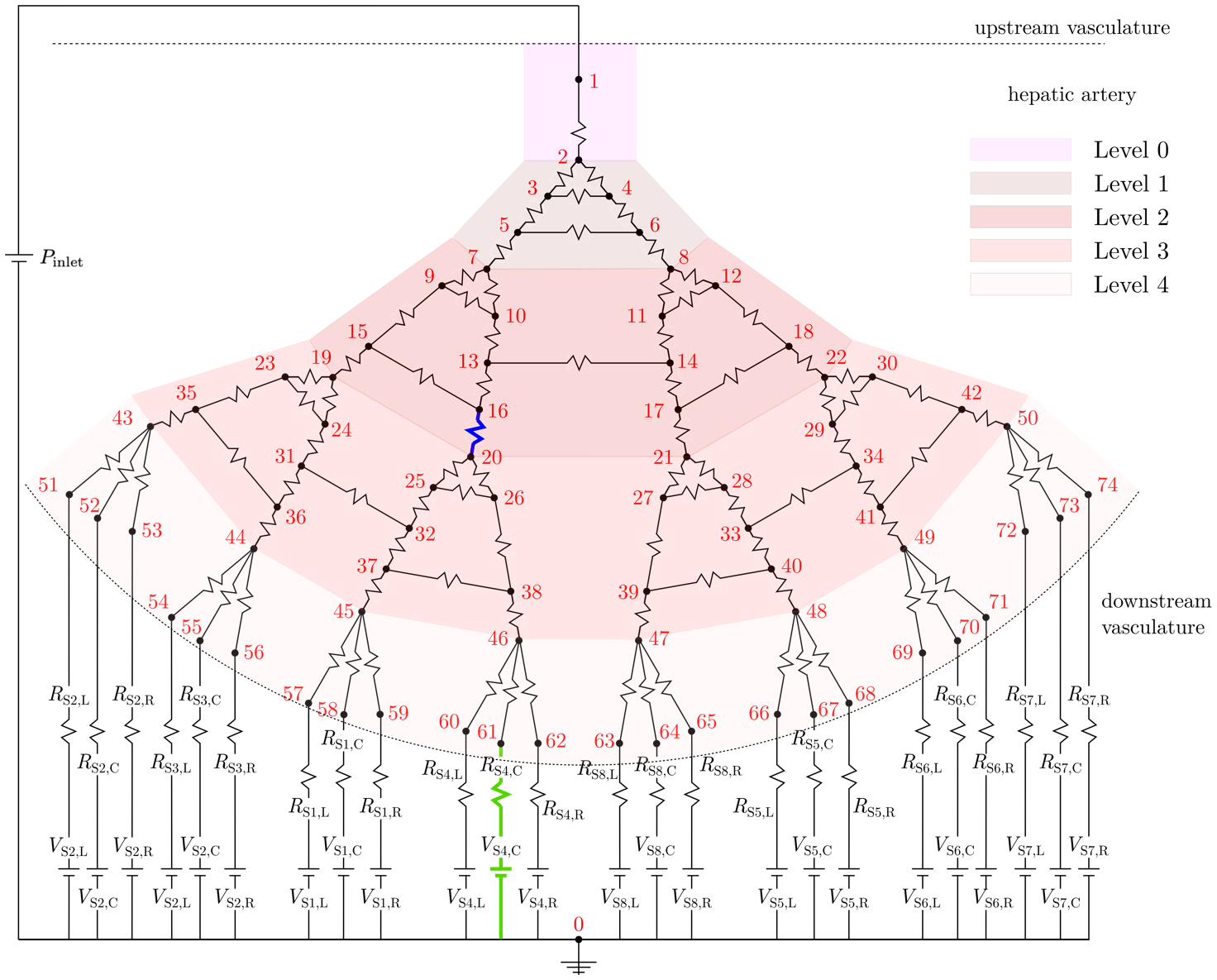


**Fig. S1** Equivalent electrical circuit (0D model) of the hepatic artery. The tumour is located in the branch (61-0), highlighted in green. The micro-balloon catheter is located in the branch (16-20), highlighted in blue.

**Table S1.** Geometrical properties of the artery branches and CAs of the 0D hepatic artery system model, showed in **Figure 2**. The length and diameter of the CAs, connecting the LHA and RHA, are 64.3 mm and 1.5 mm, respectively. The length and diameter of the CAs, connecting the segments S3-S1 and S5-S6, are 30.0 mm and 0.9 mm, respectively.

|  | **Artery Branches** | | **Upper Communicating Arcades** | | **Lower Communicating  Arcades** | |
| --- | --- | --- | --- | --- | --- | --- |
| **Level** | **Length**  **(mm)** | **Diameter**  **(mm)** | **Length**  **(mm)** | **Diameter**  **(mm)** | **Length**  **(mm)** | **Diameter**  **(mm)** |
| 0 | 80 | 5 | - | - | - | - |
| 1 | 40 | 4 | 18.9 | 1.5 | 37.7 | 1.5 |
| 2 | 30 | 3.5 | 10.0 | 1.5 | 20.0 | 1.5 |
| 3 | 28 | 3 | 9.3 | 1.5 | 18.7 | 1.5 |
| 4 | 14 | 3 | - | - | - | - |

The larger one refers to any other artery branch resistance in that level. As in Aramburu et al. (4), the upstream vasculature is modelled with a voltage (pressure) generator, $P_{inlet}=93.3$mmHg. The downstream vasculature is modelled with a resistance, $R_{Sj,X},$ and a voltage (pressure) generator, $V_{Sj,X}$, where $j=1,\ldots,8$ denotes the segment number and $X=L,C,R$, denotes the left (L), central (C), and right (R) blood vessel. The value of $R_{Sj,X}$is $571\times$10^-3^ mmHg min mL^-1^, expect for $R_{S4,C}$. As proposed by Aramburu et al. (5), the effect of the tumour is modelled by lowering the downstream resistance of the tumour-feeding artery (i.e. branch 61-0, highlighted in green in **Figure S1**) to 40% the resistance of the arteries feeding the healthy parenchyma, thus $R_{S4,C}=$228 mmHg min mL^-1^. This is because tumour-feeding arteries are usually bigger in caliber than normal hepatic vasculature. Note that the value chosen for $R_{S4,C}$ is arbitrary and it does not affect the generality of our findings. The value of $V_{Sj,X}$ is 66.66 mmHg for any downstream generator. The balloon microcatheter is located at the artery branch 16-20, that is the left daughter artery feeding the segments S1 and S4 (Level 2). When the balloon microcatheter is deflated, the resistance of the artery branch is the same as for the other branches at the same level (see **Table** **S2**). When the micro-balloon is inflated, the blood flow in the artery is obstructed and the hydrodynamic resistance of the artery is increased to simulate the artery occlusion. Practically, this is achieved by reducing the diameter of the branch 16-20 by three order of magnitudes – i.e. 99.9% of the artery cross-section is occluded by the balloon. A custom MATLAB code was used to determine the steady-state flow rates in the artery network via the loop method in case of deflated and inflated balloon (3).

**Table S2** Hydrodynamic resistance (in 10^-3^ mmHg min mL^-1^) of the hepatic artery 0D model showed in **Figure S1**. The resistance of the CA, connecting the LHA and RHA, is $R_{13,14}=$ 200$\times$10^-3^ mmHg min mL^-1^. The resistance of the CAs, connecting the segments S3-S1 and S5-S6, is $R_{31,32}= R_{33,34}=$ 719.6$\times$10^-3^ mmHg min mL^-1^. The resistance of the downstream vasculature vessels is $R_{Sj,X}=571\times$10^-3^ mmHg min mL^-1^ with $j=1,\ldots,8$ and $X=L,C,R$ – except for $R_{S4,C}=228\times$10^-3^mmHg min mL^-1^.

| **Level** | **Artery Branches** | **Upper Communicating Arcades** | **Lower Communicating Arcades** |
| --- | --- | --- | --- |
| 0 | 2.01 | - | - |
| 1 | 0.82 | 58.6 | 117.2 |
| 2 | 0.52 or 1.05 | 31.1 | 62.2 |
| 3 | 0.91 or 1.81 | 29.0 | 58.0 |
| 4 | 2.72 | - | - |

*Dye transport*

Let us consider a diluted molecular dye dispersed in a laminar flow within a straight circular vessel of radius $a$and length $L$. The cylindrical coordinates within the vessel are defined as $r\in[0,a]$ and $z\in[0,L]$. The spatio-temporal evolution of the dye concentration field, $c(r,z,t)$, is described by the time-dependent convection-diffusion equation

$$\frac{\partial c}{\partial t}+u_{z}(r) \frac{\partial c}{\partial z}=D \left[ \frac{1}{r} \frac{\partial}{\partial r}\left( r \frac{\partial c}{\partial r} \right)+ \frac{\partial^{2}c}{\partial z^{2}} \right]$$

(S3)

where $t$ is time, $D$ is the dye molecular diffusivity, $u_{z}(r)=2 U \left( 1-\frac{r^{2}}{a^{2}} \right)$ is the Hagen-Poiseuille velocity profile and $U$the flow average velocity. Eq. (S3) is completed with a set of boundary and initial conditions

$$c\left( r,0,t \right)=c_{in}\left( r,t \right)$$

$$\left. \frac{\partial c}{\partial r} \right|_{r=a}=0$$

$$c\left( r,z,0 \right)=c_{0}\left( r,z \right)$$

(S4)

with $c_{in}(r,t)$ describing the dye concentration at the inlet end of the vessel ($z=0$), and $c_{0}\left( r,z \right)$ the initial ($t=0$) distribution of dye within the vessel. The order of magnitude of the average blood velocities in hepatic arteries varies from $10 ms^{-1}$ in the PHA to $0.1 ms^{-1}$ in the segment vessels. Assuming a dye diffusivity of $5\times{10}^{-10} m^{2}s^{-1}$and a vessel diameter of $5 mm$, the resulting Peclet number $Pe=\frac{U a}{D}$ ranges from ${10}^{6}$ to ${10}^{9}.$ Consequently, the diffusive transport is negligible compared to convection and Eq. (S3) can be simplified as

$$\frac{\partial c}{\partial t}+u_{z}(r) \frac{\partial c}{\partial z}=0$$

(S5)

Eq. (S4) and (S5) can be solved independently for any flow streamline at a radial distance, $r$, from the vessel axis, via the method of characteristics. The solution can be written as

$$c\left( r,z,t \right)= \left\{ \begin{matrix} c_{in}\left( r,t -\frac{z}{u_{z}(r)} \right) \mathrm{for} t\geq\frac{z}{u_{z}(r)} \\ \\ c_{0}\left( r,z \right) \mathrm{for} t<\frac{z}{u_{z}(r)} \end{matrix} \right.$$

(S6)

The cross-section averaged dye concentration can be calculated as   $\bar{c}\left( z,t \right)=\frac{1}{\pi a^{2}}\int_{0}^{a} c(r,z,t) 2\pi r dr$. However, for sake of simplicity, we consider the dye concentration, $C\left( z,t \right),$at the flow streamline,  $r=a/\sqrt{2}$, for which the flow velocity is equal to the average velocity, i.e. $u_{z}\left( r=a/\sqrt{2} \right)=U$. By definition,

$$C\left( z,t \right)= \left\{ \begin{matrix} C_{in}\left( t -\frac{z}{U} \right) \mathrm{for} t\geq\frac{z}{U} \\ \\ C_{0}\left( z \right) \mathrm{for} t<\frac{z}{U} \end{matrix} \right.$$

(S7)

with $C_{in}\left( t \right)=c_{in}(r=a/\sqrt{2} , t)$ and $C_{0}\left( z \right)=c_{0}(r=a/\sqrt{2} , z)$. For the purpose of this study and without loss of generality, we characterize the dye concentration in a blood vessel with $C\left( z,t \right)$ instead of  $\bar{c}\left( z,t \right)$.

According to Eq. (S7), the dye concentration, $C_{ij}\left( z,t \right),$in an artery branch connecting the node $i$ and $j$ can be written as

$$C_{ij}\left( z,t \right)= \left\{ \begin{matrix} C_{i}\left( t -\frac{z A_{ij}}{q_{ij}} \right) \mathrm{for} t\geq\frac{z A_{ij}}{q_{ij}} \\ \\ C_{0,ij}\left( z \right) \mathrm{for} t<\frac{z A_{ij}}{q_{ij}} \end{matrix} \right.$$

(S8)

where $C_{i}(t)$ is the dye concentration at node $i$, $c_{0,ij}\left( r,z \right)$is the initial ($t=0$) spatial distribution of dye in the $i$-$j$ branch, $q_{ij}$ is the volumetric flow rate in the $i$-$j$ branch and $A_{ij}$ is the $i$-$j$ branch’s cross-section area. Note that Eq. (S8) assumes that the flow goes from node *i* to node *j*. Otherwise, the indices $i$ and $j$ must be swapped.

To calculate the time-evolution of dye concentration within the network, we apply the mass balance at each node of the network. Specifically, let us consider a node $k$ receiving flows from $M$ other nodes $i$, with $i$ = 1, … , M. Each node $i$ is connected to node $k$ via a branch of length $L_{ik}$. The mass balance states that $m_{in}\left( t \right)=m_{out}(t)$ with $m_{in}\left( t \right)$ the dye mass flow rate entering the node $k$ and $m_{out}(t)$ the dye mass flow rate leaving the node $k$. These rates are calculated as

$$m_{in}\left( t \right)=\sum_{i=1}^{M} m_{ik}\left( t \right) , m_{out}\left( t \right)=C_{k}\left( t \right) \sum_{i=1}^{M} q_{ik}$$

(S9)

where $m_{ik}\left( t \right)$ is the dye mass flow rate entering node *k* from node *i* through the $i$-$k$ branch. According to Eq. (S8), this mass flow rate can be expressed as

$$m_{ik}\left( t \right)= \left\{ \begin{matrix} q_{ik} C_{i}\left( t -\frac{L_{ik} A_{ik}}{q_{ik}} \right) \mathrm{for} t\geq\frac{L_{ij} A_{ik}}{q_{ik}} \\ \\ q_{ik}C_{0,ik}\left( L_{ik}-\frac{q_{ik} t}{A_{ik}} \right) \mathrm{for} t<\frac{L_{ij} A_{ik}}{q_{ik}} \end{matrix} \right.$$

(S10)

By combining the mass balance and Eq. (S9), one gets

$$C_{k}\left( t \right)=\frac{\sum_{i=1}^{M} m_{ik}\left( t \right)}{\sum_{i=1}^{M} q_{ik}}$$

(S11)

This expression together with Eq. (S10) relates the concentration at node *k* at time $t$ to the concentrations at nodes $i$ at previous times. Hence, these relations can be used to calculate the time evolution of the concentrations at any nodes of the network, once the initial ($t=0$) dye distributions, $C_{0,ij}(z)$, is defined for any $ij$ branch of the network. Numerically, this can be done by expressing Eq. (S11) at discrete time points $t_{n}=n \Delta t$, so that

$$C_{k}\left( t_{n} \right)=\frac{\sum_{i=1}^{M} m_{ik}\left( t_{n} \right)}{\sum_{i=1}^{M} q_{ik}}$$

(S12)

and

$$m_{ik}\left( t_{n} \right)= \left\{ \begin{matrix} q_{ik} C_{i}\left( t_{n-p} \right) \mathrm{for} t_{n}\geq\frac{L_{ij} A_{ik}}{q_{ik}} \\ \\ q_{ik}C_{0,ik}\left( L_{ik}-\frac{q_{ik} t_{n}}{A_{ik}} \right) \mathrm{for} t_{n}<\frac{L_{ij} A_{ik}}{q_{ik}} \end{matrix} \right.$$

(S13)

with $p=\mathrm{round}\left( \frac{L_{ik} A_{ik}}{q_{ik} \Delta t} \right)$.

A custom Matlab code was developed to calculate the spatio-temporal distribution of dye in the artery network. To simulate the dye injection from the balloon microcatheter located at node 20, Eq. (S12) and Eq. (S13) are used with the initial conditions $C_{20}\left( t=0 \right)=1$ and $C_{0,ij}\left( z \right)=0 \forall i,j$. The time step $\Delta t$ is calculated as $0.1\times\min_{i,j} \left( \frac{L_{ij} A_{ij}}{q_{ij}} \right)$. Once the time evolution of the node concentrations, $C_{k}\left( t_{n} \right)$, are known for any $k$ and $n$, Eq. (S8) can be used to calculate the spatio-temporal evolution of dye in all branches of the network. Numerically, this can be done by expressing Eq. (S8) at discrete time points $t_{n}$ and spatial points $z_{m}=m \Delta z$,

$$C_{ij}\left( z_{m},t_{n} \right)= \left\{ \begin{matrix} C_{i}\left( t_{n-p} \right) \mathrm{for} t\geq\frac{z_{m} A_{ij}}{q_{ij}} \\ \\ C_{0,ij}\left( z_{m} \right) \mathrm{for} t<\frac{z_{m} A_{ij}}{q_{ij}} \end{matrix} \right.$$

(S14)

with $p=\mathrm{round}\left( \frac{z_{m} A_{ik}}{q_{ik} \Delta t} \right)$. The spatial step for the $i$-$j$ branch is calculated as $\Delta z=L_{ij}/50$. The video included in the Supplementary Information file was generated in Matlab.

Finally, to quantify the efficacy of b-TACE treatment, it is useful to calculate the percentage of dye mass, ejected from the catheter, that reaches the tumour as

$$m_{\%}\left( t \right)=\frac{M_{tumor}\left( t \right)}{M_{catheter} \left( t \right)}\times100$$

(S15)

where $M_{catheter}\left( t \right)$ is the total mass of dye ejected from the balloon microcatheter and $M_{tutor}\left( t \right)$ is the total mass of dye received by the tumour within the time range $\left[ 0,t \right].$ Both $M_{catheter}\left( t \right)$ and $M_{tutor}\left( t \right)$ can be calculated from the total mass of dye that has flowed through the arteries connected to the catheter and tumour, respectively. The total mass of dye that has flowed through any artery branch $i$-$j$ between times 0 and $t$ can be calculated as

$$M_{ij}(t)= \left\{ \begin{matrix} \int_{0}^{t} C_{i}\left( \tau\right) q_{ij} d\tau\mathrm{for} q_{ij}\geq0 \\ \\ \int_{0}^{t} C_{j}\left( \tau\right) q_{ij} d\tau\mathrm{for} q_{ij}<0 \end{matrix} \right.$$

(S16)

**REFERENCES**

1. Oh KW, Lee K, Ahn B, Furlani EP. Design of pressure-driven microfluidic networks using electric circuit analogy. Lab Chip. 2012;12(3):515–45.

2. Jr JRB, Kleinstreuer C, Comer JK. Rheological eects on pulsatile hemodynamics in a stenosed tube. 2000;30.

3. Aramburu J, Antón R, Rivas A, Ramos JC, Larraona GS, Sangro B, et al. Numerical zero-dimensional hepatic artery hemodynamics model for balloon-occluded transarterial chemoembolization. Int J Numer Methods Biomed Eng. luglio 2018;34(7):e2983

4. Aramburu J, Antón R, Rivas A, Ramos JC, Larraona GS, Sangro B, et al. Numerical zero-dimensional hepatic artery hemodynamics model for balloon-occluded transarterial chemoembolization. Int J Numer Methods Biomed Eng. luglio 2018;34(7):e2983

5. Aramburu J, Antón R, Fukamizu J, Nozawa D, Takahashi M, Ozaki K, et al. In Vitro Model for Simulating Drug Delivery during Balloon-Occluded Transarterial Chemoembolization. Biology. 16 dicembre 2021;10(12):1341.
